# Investigating the Constraints and Mitigation Strategies for the Adoption of Sustainable Land Management Practices in Erosion-prone Areas of Southeast Nigeria

**DOI:** 10.1007/s00267-024-02104-y

**Published:** 2025-01-15

**Authors:** Cynthia Nneka Olumba, Guy Garrod, Francisco Areal

**Affiliations:** School of Natural and Environmental Sciences, Newcastle University, Newcastle-upon-Tyne, UK

**Keywords:** Adoption, Farmers, Constraints, Recommendations, Sustainable land management practices, Land degradation

## Abstract

The adoption of sustainable land management practices (SLMPs) is crucial to improve soil health, and farm yield, and potentially limit the degradation of agricultural and ecological systems. However, farmers still encounter diverse challenges when trying to implement SLMPs. Research on the potential mitigation strategies to address the complex challenges to the adoption of SLMPs in the developing countries context is limited. Accordingly, this study investigates the constraints to adopting SLMPs using household survey data collected from 480 sampled farmers in erosion-prone areas of southeast Nigeria. Also, through focus groups and interviews with key stakeholders in the land sector, the study investigates the potential mitigation strategies to address the constraints. Descriptive statistics were used to explore the characteristics of the farmers, while Principal component analysis (PCA) was used to analyse the constraints to the adoption of SLMPs. The qualitative data collected were analysed using inductive thematic analysis. The PCA result identified economic/financial factors as the principal constraint to the adoption of SLMPs. Other barriers to the adoption of SLMPs in the study area include constraints related to the characteristics of the SLMPs, institutional constraints and constraints related to land property rights. Based on the stakeholders’ perspectives, financial and economic support, improved R&D, knowledge exchange and advisory system, policy and regulatory solutions, and multi-stakeholder engagement are important strategies to tackle the constraints to the adoption of SLMPs. Insights from this study could help practitioners, conservation planners and policymakers design more targeted and effective interventions to promote the widespread adoption of SLMPs.

## Introduction

Globally, the use of sustainable land management practices (SLMPs) has been recognized as a suitable solution to improve agricultural land productivity, protect soil and water resources, and provide a profitable and socially appealing solution to land degradation problems (Ruiz et al. 2020). As agricultural land stewards, farmers across the globe are encouraged to shift from traditional, unsustainable farming methods to implement SLMPs to abate agricultural land degradation. However, previous studies have shown that farmers’ adoption and continuous use of SLMPs is challenging due to various barriers faced by farmers. One often cited constraint to adopting SLMPs is land tenure security. Decision-making about investment in SLMPs (e.g., agroforestry and terracing) often requires longer-term investments and without the security of land tenure, farmers are discouraged from adopting SLMPs (Olumba et al. 2024b; Rahman et al. 2017).

Moreover, implementing many SLMPs often requires high initial investments and additional operational and maintenance costs (such as the costs of maintaining terrace structures and the additional labour for weeding after implementation). Smallholder farmers in many developing countries often do not have enough money to cover the costs of implementing SLMPs (Rahman et al. 2017). Similarly, the financial demands of SLMPs can be a barrier to adoption for farmers in developed regions (Mills et al. 2020). Additionally, farmers’ adoption of SLMPs is limited by the lack of economic incentives such as subsidies on agricultural inputs or other forms of government support, especially in developing economies (Grabowski 2011; Osmond et al. 2015). In developed economies, where incentives are available, the high level of bureaucracy involved in obtaining agricultural subsidies often discourages farmers’ access to these incentives to support their adoption of SLMPs (Marques et al. 2015; Reimer and Prokopy 2014). Several other factors constrain farmers’ adoption of SLMPs including farmers’ lack of knowledge and expertise of implementing SLMPs (Barbosa Junior et al. 2022; Toth et al. 2017), poor institutional arrangements for information delivery and credit supply (Cholo et al. 2018; Marques et al. 2015; Olumba et al. 2023), and market-related factors (Andersson and D’Souza 2014; Mills et al. 2020). Based on the existing literature, it is hypothesised that a variety of market and institutional factors constrain farmers’ adoption of SLMPs including socio-economic and financial factors along with a lack of government support combined with high levels of bureaucracy. Hence, the over-arching objectives of the study are to:

1. identify the key constraints to the adoption of SLMPs.
2. identify stakeholders’ perspectives on the mitigation strategies to address the constraints to the adoption of SLMPs.

This study was conducted in Anambra and Imo states, identified as erosion-prone areas in the southeast region of Nigeria (Tochukwu 2022). The areas’ vulnerability to erosion is primarily attributed to the characteristics, topographical features, and geological composition of the soils in the area. As documented in the literature, the soils in the southeast region of Nigeria are structurally unstable and thus have a high susceptibility to erosion and a high rate of runoff (Okorafor et al. 2017; Tochukwu 2022). Ufot et al. (2016) further emphasised that the soils in the area are characterised by low organic matter content and water storage capacity, making them highly susceptible to erosion activities. Moreover, other factors including the high rainfall intensity in the region and poor land use activities like unsustainable farming methods, deforestation, and overgrazing further exacerbate soil erosion problems in the region (Okereke and Emeribeole 2020). The predominant type of erosion in the study area is gully erosion, with scholars reporting about 450 and 700 gully sites in the Imo and Anambra states respectively (Okereke and Emeribeole 2020).

Given the enormous challenges posed by soil erosion in the region and its adverse consequences on farming systems, this study is particularly relevant. Moreover, this study makes important contributions to the extant literature on SLMPs adoption in several ways. First, most of the past studies on farmers’ adoption of SLMPs rely on regression models to characterize factors influencing adoption typically, demographic characteristics of the farmers such as age, level of education, gender, and income (Mishra et al. 2018; Olumba and Olumba 2024; Olumba et al. 2024b). However, there is a notable lack of research focused on the challenges that farmers encounter when trying to implement SLMPs, especially in developing countries. We argue that it is important to look beyond just identifying the drivers of adoption. A deeper exploration into the specific challenges that farmers encounter when trying to implement SLPMs is essential to identify key areas for policy intervention. Secondly, existing studies on the barriers to adopting SLMPs often fail to thoroughly explore stakeholders’ views on strategies to overcome the adoption barriers (e.g., Jellason et al. 2021; Toth et al. 2017). To address the above research gaps, this study aimed to investigate the constraints hindering the adoption of SLMPs and to explore possible strategies for mitigating these challenges. The findings of this study can guide practitioners, conservation planners and policymakers in designing more targeted and effective interventions to promote the widespread adoption of SLMPs in the area and in Nigeria generally. The societal implications of this study include its contribution to the achievement of several United Nations Sustainable Development Goals (SDGs) including SDG 1 (No poverty), SDG 2 (Zero hunger), SDG 13 (climate action) and SDG 15 (life on land). By addressing the barriers and fostering an enabling environment for farmers, developing countries such as Nigeria can improve the rate of adoption of SLMPs which can enhance agricultural productivity, improve soil health, ensure food security and the long-term viability of local farming communities.

## Methodology

### Study Area

**Fig. 1:**
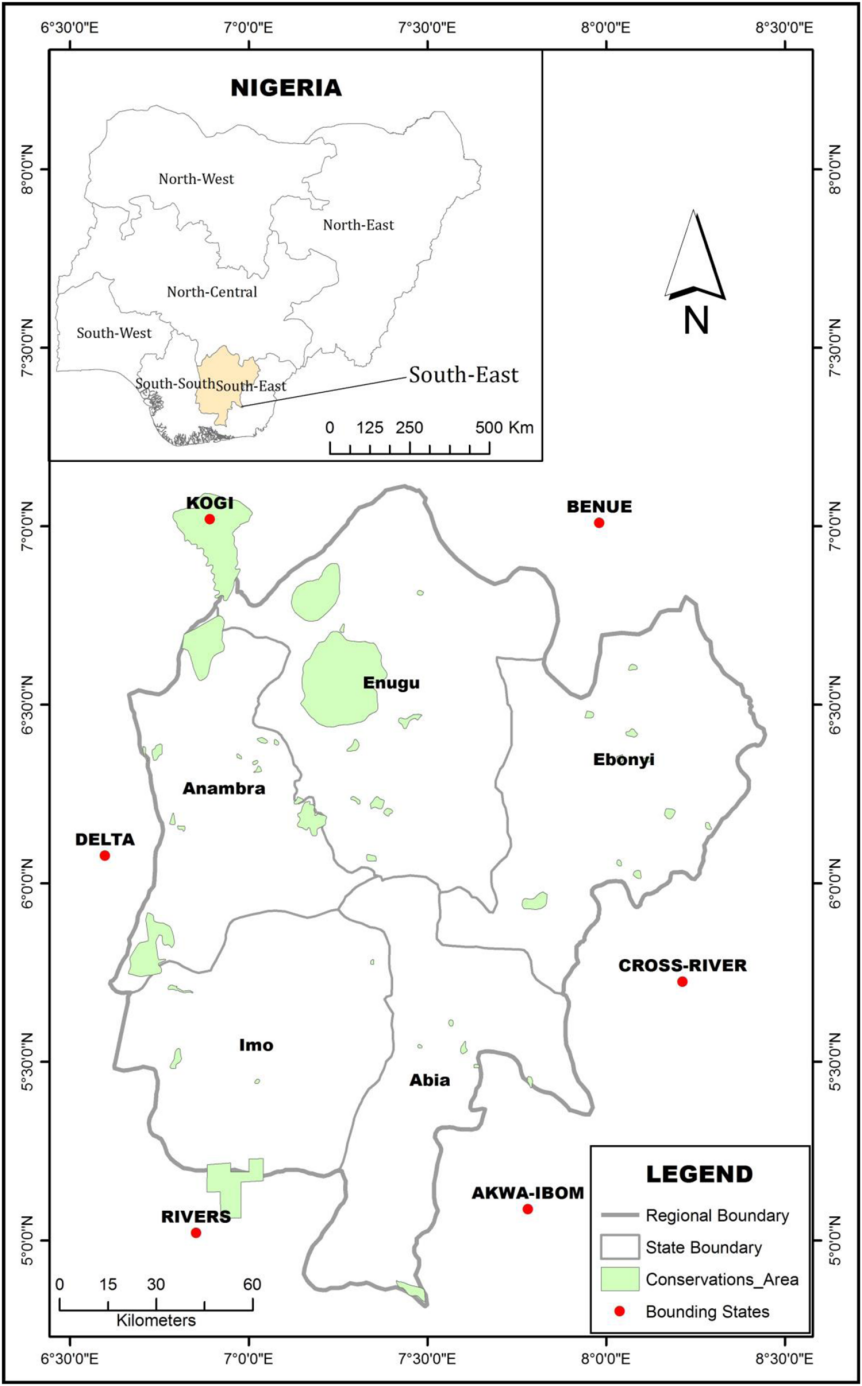
Map of the Southeast region of Nigeria showing the study locations. Note: The study locations are denoted by purple circles. Map of Nigeria and Southeast Nigeria is inset. Source: Olumba et al. (2024a)

Anambra and Imo states are situated in the southeast region of Nigeria (Fig. 1). Anambra and Imo states are located between approximately 5° 11’ 56” and 6° 45’ 07” North, and between approximately 6° 37’ 53” and 7° 25’ 39” East respectively. According to the 2006 population census, Anambra and Imo states have a population of 4,182,032 and 3,934,899 residents respectively (Tochukwu 2022). The area’s tropical rainforest climate allows farming activities including food crop production and livestock rearing to thrive. The majority of smallholder farmers in the region rely on farming for their livelihoods. However, farming activities in the area are significantly affected by land degradation, largely due to the persistent problem of soil erosion (Ndulue et al. 2021).

### Research Design

The study combined a mixed-method approach with a multi-method approach. By using a mixed method design the study integrates the findings from focus groups and interviews (qualitative analysis) and household surveys (quantitative analysis), resulting in a deeper understanding of the constraints encountered by farmers in implementing SLMPs—research objective 1. The multi-method design (i.e. the use of multiple qualitative data obtained from focus groups and interviews), allowed the thorough investigation of the stakeholders’ perspective on the strategies to mitigate the challenges hindering the adoption of SLMPs—research objective 2. Utilising mixed and multimethod designs in this present study, allowed the analysis of the research objectives from multiple perspectives, providing a more comprehensive understanding of the phenomenon under investigation. Pashaie et al. (2023) assert that when researchers employ multiple research methods simultaneously, taking into account various approaches to data collection and interpretation, the research outcomes become more reliable.

#### Qualitative Study—Methods of Data Collection and Sources

The qualitative research entailed conducting 25 semi-structured interviews (both in-person and over the phone). Only the interviews with the farmers were done in person, all other interviews were done remotely. The remote interviews were mainly motivated by the travel restrictions caused by the COVID-19 pandemic at the time of research. Interviewees were recruited through snowball sampling (Cohen et al. 2011). Table 1 presents the information on the interview participants. The interview guide used to conduct the interviews is published elsewhere (Olumba et al. 2024a). Interviewing this range of stakeholders was important for this study to gather more information and diverse perspectives from their varied fields of expertise. An interview guide designed to facilitate the interviews contained questions that investigated the prominent challenges faced by farmers in their adoption of SLMPs and solutions to address these challenges. The flexible structure of the interview guide enabled the development of follow-up and probing questions to ensure that each participant’s account was fully explored (Knox and Burkard 2009).

**Table 1:** Details about the interview participants

Participants group	Organisation	Number	Participant number
Scientific Community	Resource and Environmental Policy Research Centre, (REPRC) Environment for Development^a^	2	P1-P2
Agricultural- and environmental-based NGOs	Foundation for Livelihood Advancement	3	P3-P5
	Sasakawa-Global 2000-Nigeria		
	The Nigerian Environmental Study Action Team (NEST)^b^		
State Agricultural Development Programme (ADP)	State Ministry of Agriculture and Rural Development	1	P6
	Women in Agriculture	3	P7-P9
	Extension Agents	4	P10-13
	ADP staff	2	P14-15
Farmers	Key informant farmers	10	P16-P25
Total		**25**	

^a^The Resource and Environmental Policy Research Centre (REPRC) is a research centre established in 2018 by the University of Nigeria Nsukka. The centre has a core aim of tackling environmental and development challenges through policy-relevant research and policy engagement

^b^The Nigerian Environmental Study Action Team (NEST) is a national-level non-government organisation with a core aim of addressing the issues of the environment and sustainable development through conducting research to inform policy and stakeholder action

In addition to the interviews, 6 focus group sessions were conducted in person with farmers. The number of focus group sessions is above the recommended minimum of three to four sessions for simple research topics (Burrows and Kendall 1997). It is important to mention that in line with the saturation principle for determining the number of effective focus group sessions (Krueger 2014), while four focus group sessions were conducted in Anambra state, only two focus group sessions were held in Imo state. This is because no new information was generated from the sessions compared to the information previously gathered from the sessions in Anambra state. To obtain rich and important data, focus group participants comprised farmers still practising, or who had previously practised SLMPs. The main aim of the sessions was to discuss the main challenges to adopting SLMPs and options for tackling them. In the interest of brevity, information on the focus group participants is reported in Appendix 3. The focus group participants were recruited with the assistance of the extension agents (EAs) working at the Agricultural Development Programme (ADP) office in both states.

#### Quantitative Study- Methods of Data Collection and Data Sources

The quantitative research involved a household survey of 480 sampled farmers. A four-stage sampling technique comprising purposive and simple random sampling was employed to select the sampled farmers: 240 farmers from Anambra and 240 farmers from Imo state for the study (see Appendix 1). The quantitative data for this research were obtained using a structured questionnaire (see Appendix 2). A pilot test of the questionnaire was carried out with non-sampled farmers. This process helped to identify and clarify ambiguous questions in the questionnaire and also allowed the research teams to get acquainted with the questionnaire. The questionnaire was modified following the pilot survey. The pre-tested questionnaire was uploaded to the Qualtrics platform and administered using tablets loaded with the Qualtrics app. Due to the low literacy level of farmers in the area, the questionnaire was administered via face-to-face interviews. Also, given that the survey was conducted in areas where internet access was often poor, responses were saved on the tablets and uploaded to the Qualtrics server as soon as an internet connection became available.

The questionnaire collected information on the socio-economic characteristics of the farmers, their adoption of SLMPs, and constraints to the adoption of SLMPs. A list of constraints to the adoption of SLMPs was presented to the farmers, they were then asked to indicate the level of constraints faced when adopting or considering adopting SLMPs using a Likert scale, ranging from very low extent (equal to a score of 1) and very great extent (equal to a score of 4). The forced-response option on Qualtrics was applied to the questions; this ensured that the dataset had no missing values. The items on the list were obtained from related existing literature (Jellason et al. 2021; Kohio et al. 2017). To facilitate the research and get maximum response rates, four research assistants were recruited and trained on the contents of the questionnaire, how to administer the questionnaire and any confidentiality issues that need to be considered while conducting the survey. Ethical approval for this study was granted by the Research Ethics Committee of the Newcastle University. After obtaining the participant’s consent, all interviews and focus group discussions were audio-recorded and later transcribed for analysis. In the result section, quotations from the transcripts are referenced using the participants’ numbers.

### Data Analysis

The qualitative data collected were transcribed verbatim and analysed through inductive thematic analysis. The thematic analysis was supported using Nvivo version 12 software following an iterative and reflexive process (Bryman 2016). The iterative and reflexive process involved reading and rereading all interview data and using the emerging codes to develop themes and sub-themes. See Appendix 4 for a reflection on the codebook generation. Also, an overview and description of the themes and sub-themes are provided in Table A1 in the Appendix.

For the quantitative analysis, descriptive statistics was used to explore the farmers’ characteristics and the adoption pattern of SLMPs. Principal component analysis (PCA) was used to analyse the constraints to the adoption of SLMPs among the sampled farmers. The quantitative analysis was carried out using IBM SPSS version-27 analytical software. For triangulation purposes, the qualitative research findings were used to validate and explain the results of the PCA analysis (Bryman 2016).

#### The PCA Model

Following the procedures of Olumba et al. (2021), the PCA was employed to analyse the constraints to the adoption of SLMPs. PCA was carried out to statistically summarize a set of interrelated variables Xi’s (i = 1, 2, …, j) by decreasing the number of variables in the dataset to a reduced set of dimensions (principal components). These principal components (Y1……Yn) represent a linear combination of the X’s as specified in equation 1 below:

The PCA model is stated as follows:

$$\begin{array}{lll}{\rm{Y}}1 \, = \, {{\rm{a}}}_{11}{{\rm{X}}}_{1}+{{\rm{a}}}_{12}{{\rm{X}}}_{2}+{{\rm{a}}}_{13}{{\rm{X}}}_{3}+{{\rm{a}}}_{14}{{\rm{X}}}_{4}\ldots \ldots .{{\rm{a}}}_{1{\rm{j}}}{{\rm{X}}}_{{\rm{j}}}\\ {\rm{Y}}2 \, = \, {{\rm{a}}}_{21}{{\rm{X}}}_{1}+{{\rm{a}}}_{22}{{\rm{X}}}_{2}+{{\rm{a}}}_{23}{{\rm{X}}}_{3}+{{\rm{a}}}_{24}{{\rm{X}}}_{4}\ldots \ldots .{{\rm{a}}}_{2{\rm{j}}}{{\rm{X}}}_{{\rm{j}}}\\ {\rm{Y}}3 \, = \, {{\rm{a}}}_{31}{{\rm{X}}}_{1}+{{\rm{a}}}_{32}{{\rm{X}}}_{2}+{{\rm{a}}}_{33}{{\rm{X}}}_{3}+{{\rm{a}}}_{34}{{\rm{X}}}_{4}\ldots \ldots .{{\rm{A}}}_{3{\rm{j}}}{{\rm{X}}}_{{\rm{j}}}\\ \ldots \ldots \ldots \ldots \, \, \ldots \ldots \,\ldots \ldots \ldots \, \ldots \ldots \,\ldots \ldots \ldots ..\\ \ldots \ldots \ldots \, \, \ldots \ldots \ldots \,\ldots ..\ldots \, \ldots \ldots \ldots .\,\ldots \ldots \ldots .\\ {\rm{Yn}} \, = \, {{\rm{a}}}_{{\rm{j}}1}{{\rm{X}}}_{1}+{{\rm{a}}}_{{\rm{j}}2}{{\rm{X}}}_{2}+{{\rm{a}}}_{{\rm{j}}3}{{\rm{X}}}_{3}+{{\rm{a}}}_{{\rm{jj}}}{{\rm{X}}}_{{\rm{j}}}\end{array}$$

Where Y1, Y2 …, and Yn represent the principal components to be derived from the analysis of data. X1, X2, X3, and X4 represent the observed variables/ constraints to the adoption of SLMPs; a_1_ – a_4_ represent the correlation coefficients or factor loadings. The as, called loadings are chosen based on the conditions that the constructed principal components are not correlated, and that Y1 explains most of the variability in the set of all Xs. Also, Y2 explains the maximum of the remaining variation in the Xs (after adjusting for the variation captured by Y1, etc.) (Koutsoyiannis 1977).

## Results

### Socio-economic Characteristics of the Sampled Farmers

This section presents the descriptive statistics of some key socioeconomic variables of the farmers. As shown in Table 2, about 61% of the farmers are female. The mean age of the sample is approximately 51 years. The average farm income earned in the previous farming season was approximately ₦155,182 (equivalent to US$364.71, August 2022).

**Table 2:** Descriptive statistics of the characteristics of the sampled farmers (*N* = 480)

Characteristics of the farmer	Frequency	Percentage	Mean
**Gender**			
Male	188	39.2	
Female	292	60.8	
**Age (years)**			50.66
30–39	110	22.9	
40–49	88	18.3	
50–59	99	20.6	
60–69	97	20.2	
>69	61	12.7	
**Total farm income in the last farming season (₦)**			155,182.29
<100,000	204	42.5	
100,000–150,000	132	27.5	
151,000–200,000	57	11.9	
201,000–300,000	50	12.6	
301,000–400,000	11	11	
>400,000	26	2.4	
**Farm size in hectares**			1.05
<2 hectares	414	86.3	
2–3.9 hectares	50	10.4	
4–5.9 hectares	5	1	
6 to 9.9 hectares	5	1.0	
10–15 hectares	6	1.3	
**Tenure status of main farmland**			
Communal land	24	5	
Sharecropping	4	0.8	
Rented	138	28.8	
Private/purchased	23	4.7	
Inheritance	271	56.5	
Farming on land for free	20	4.2	
**Tenure Document (if farmers have formal or informal land tenure documents)**			
Yes	128	26.7	
No	352	73.3	
**Credit access**			
Formal institutions e.g. micro-finance bank	16	3.3	
Informal institutions e.g. cooperatives, friends, and relatives	123	25.6	
I need credit but do not have access to credit	167	34.8	
I do not need credit	174	36.3	
**Number of Extension visits in last year**			1.04
0	359	74.7	
1	57	11.9	
2	25	5.2	
3	16	3.3	
4	4	0.8	
5–10	15	3.1	
11–15	2	0.4	
16–20	1	0.2	
20–25	1	0.2	

Most of the sampled farmers (86%) are smallholders, cultivating an average of one hectare of farmland. Table 2 also shows that the major mode of farm acquisition in the area is through inheritance (56.5%), followed by rental (28.8%). Only about 5% of farmlands were acquired through outright purchase. 4.2% of farms were temporarily acquired for farming from family members at no cost. The result shows that less than a third (27%) of the plots have formal or informal documents confirming their tenure. This observed poor rate of land registration is also reported by Edeh et al. (2022).

Furthermore, over a third (34.8%) of the farmers indicated that they need credit but do not have access to it. 25.6% of the farmers indicated that they have access to credit and acquire it through informal sources, such as cooperatives, friends, and relatives. Only 3.3% of the farmers reported obtaining credit from formal financial institutions, such as microfinance banks, suggesting that farmers in the study area rarely turn to commercial banks for financial support. Meanwhile, 36.3% reported that they did not think they needed credit, and thus were not interested in applying it. Furthermore, the vast majority (74.7%) of the farmers had had no extension contact(s) (face-to-face or phone) in the last year, with an average of only one contact.

### Adoption Pattern of the Existing SLMPs in the Area

Table 3 shows the adoption pattern of the existing SLMPs in the area. The table shows that land fallow practices (87%), agroforestry (73%) and crop residue management (72%) were the three most adopted SLMPs. The two least adopted practices were minimum tillage and terracing, implemented by only 10% and 13% of the farmers respectively. The contribution of these SLMPs to tackling agricultural land degradation is discussed elsewhere (Olumba et al. 2024a).

**Table 3:** Frequency distribution of the existing SLMPs adopted by the farmers (*N* = 480)

SLMPs	Frequency	Percentage
**Agroforestry**		
Yes	349	72.7
No	131	27.3
**Crop residue management (CRM)**		
Yes	346	72.08
No	134	27.92
**Terracing**^**a**^		
Yes	62	12.9
No	127	26.5
**Contour Farming**^**a**^		
Yes	107	22.3
No	82	17.1
**Minimum Tillage**		
Yes	50	10.4
No	430	89.6
**Land fallow**		
Yes	421	87.71
No	59	12.29
**Integrated Soil Fertility Management (ISFM)**		
Yes	221	46
No	259	54

^a^The total does not sum to 480, due to omitted responses (*n* = 291) from farms with flat/no slope

### Constraints to the Adoption of SLMPs Among the Sampled Farmers

In this section, we integrate the results from the qualitative data (focus group and interviews) with the results from the quantitative survey, recognising the strength of mixed-method research (Mackey and Bryfonski 2018). Table 4 shows the results of the varimax-rotated PCA analysis. The analysis of data showed that the model fit for the PCA was satisfactory. The PCA yielded an acceptable Kaiser-Meyer-Olkin (KMO) value of 0.88 which was higher than the 0.50 threshold (Kaiser, 1974), and the Bartlett test of sphericity was statistically significant (*p* < 0.001), thus confirming that the dataset is suitable for PCA (Kissi et al. 2017). The Kaiser (1958) criterion was applied to obtain the factor loadings. Following Kissi et al. (2017), factor loadings with absolute values greater than 0.50 were set as the threshold for selecting the underlying factors or principal components explaining the data. All 17 constraint items were found to be categorised into four distinct factors which represent the major issues limiting the adoption of SLMPs in the study area. All four factors have eigenvalues higher than 1 and together account for 63.78% of the variability in the dataset, fulfilling the minimum percentage of 60% argued by Malhotra et al. (2006).

**Table 4:** PCA result on the constraints to the adoption of SLMPs in Southeast Nigeria

	Factor 1	Factor 2	Factor 3	Factor 4
High prices of agricultural inputs (e.g., herbicides, fertilizers, manure)	0.681			
Untimely/inconsistent supply of inputs by the government	0.883			
Lack of economic incentives in terms of government support for agricultural input subsidies (seed and fertilizer), and farm implements.	0.894			
High cost of SLMPs materials (e.g manure or improved seed varieties)	0.847			
High cost of labour	0.755			
Lack of finance to implement SLMPs	0.826			
Failure of previous SLMPs implemented		0.824		
Temporal delays in realising the benefits of SLMPs		0.696		
Competing use of mulch materials/crop residue for livestock feed or firewood		0.669		
Time demands of implementing SLMPs		0.559		
Use of mulch materials/ crop residue could attract pests/rodents/mice to my farm		0.523		
Lack of access to SLMPs information			0.757	
Lack of access to credit from formal/informal financial institutions at the time needed and in sufficient quantities			0.536	
Lack of advisory support from extension agents			0.697	
Lack of access to land at the time needed and in sufficient quantities				0.608
The fear that the land can be taken from you at any time				0.654
Uncontrolled grazing by livestock that feeds on crop residue on farm				0.760
Percentage (%) of the total variance	35.89	14.30	7.61	5.97
Cronbach’s alpha (α)	0.913	0.744	0.760	0.51
Composite reliability	0.923	0.792	0.705	0.787

Regarding research objective 1, the PCA result suggests four main constraints to the adoption of SLMPs in the area. Factor 1 which is the most influential factor represents economic/financial constraints. Variables that dominated this factor are: high prices of agricultural inputs; the untimely/inconsistent supply of inputs by the government; a lack of economic incentives in terms of government support for farm input subsidies; the high cost of SLMPs materials; the high cost of labour; and a lack of finance to implement SLMPs. Factor 2 represents constraints related to the peculiar characteristics of certain SLMPs. The loadings under this second factor are: failure of previously implemented SLMPs; temporal delays in realising the benefits of SLMPs; competing use of mulch materials/crop residues for livestock feed or firewood; the time demands for implementing SLMPs; and the concern that the use of mulch materials/crop residues will attract pests to the farm.

Factor 3 represents institutional constraints. The loadings under the third factor are: a lack of access to information on SLMPs; a lack of access to credit from formal/informal institutions when needed and in sufficient quantities; and a lack of advisory support from extension agents. Factor 4 represents constraints related to land property rights. The loadings under the fourth factor are: lack of access to land when needed and in sufficient quantities; the fear that the land could be taken from you at any time; and the threat of uncontrolled grazing by livestock that feed on crop residues on the farm.

#### Factor 1: Economic/Financial Constraints

The lack of government support in terms of the timely provision and consistent supply of subsidised agricultural inputs (e.g., seeds, fertilizer, and farm implements) to incentivise farmers, emerged as a major economic constraint to the adoption of SLMPs. The results from the qualitative study support this survey findings. For example, P21 stated: “*Farm inputs from the government do not always come. Whenever the inputs arrive, it is* *usually late”*. Similarly, P23 stated: “*In general, the government does not support us by providing fertilizer and other farming resources for us*”.

Another highlighted constraint to SLMP adoption in the area relates to the high cost of agricultural inputs (e.g., manure, fast-yield tree seedlings and farm labour), and the lack of finance to implement SLMPs (e.g., labour costs to implement terraces). Again, the qualitative findings corroborate this PCA result. During his interview, P14 highlighted how farmers limited financial capacity impedes their adoption of agroforestry: “*Farmers will tell you that they are poor and cannot afford the cost of improved seed varieties of Ukwa [local economic tree] that matures early and gives higher yield*”. Moreover, the farmers’ focus group revealed a general concern about the high cost of organic fertilisers which limits the adoption of ISFM. For example, one of the farmers stated: “*It [manure] is costly; It is not everybody that can afford it, so ISFM is not a common practice here*” (mixed-gender focus group, P6, Imo state).

#### Factor 2: Constraints Related to the Characteristics of the SLMPs

The result of the PCA suggests that the temporal characteristics of SLMPs, e.g., the length of time it takes to earn profit from agroforestry, act as major constraints to their adoption. From this perspective, P16 expressed the following concerning agroforestry practice: “*You know that tree planting takes time to produce; in our area, we need something that we can just take to the market after harvest and get the money quickly*”. Additional factors constraining SLMP adoption, according to the farmers, include the time investment required to ensure that SLMPs are firmly established (e.g., time to construct terraces). Moreover, the farmers sampled in this study indicated that the scarcity of crop residues due to their competing use as animal feed, or as firewood for household cooking often discourages them from implementing crop residue management. The farmers also indicated that keeping crop residue on their farms attracts pests to their farms and this situation discourages their practice of crop residue management. The qualitative findings corroborate the PCA result. For example, during the focus group, a farmer stated: *“whenever I leave the residue of harvested crops on my land, it seems to attract pests that badly affect my crops. So even though I know it is good, I am a bit hesitant to do so” (mixed-gender focus group, participant 8, Anambra state).* Another focus group participant stated*: “my farm size is small, the maize residues available are not enough to feed my goats and sheep. This shortage makes it difficult to practice crop residue management on my farm” (female-only focus group participant 1, Anambra state)*. Additionally, the PCA result shows that the situation where SLMPs fail to realise expected yield improvements also served as a deterrent to farmers’ adoption of SLMPs.

#### Factor 3: Institutional Constraints

The PCA result suggests that the lack of access to information about SLMPs and access to advisory support from EAs in the study area challenges the adoption of SLMPs. The results from the qualitative study support this PCA result. During the focus group, a farmer stressed the importance of receiving guidance from EAs before adopting crop residue management which she perceived as very beneficial but challenging to adopt due to the risks of mismanagement. According to her:

*“if you go into crop residue management without the advice of someone who knows how to do it, it might not end well, especially if the residues are not adequately incorporated into the soil. Also, you can suffer from pest infestation and your yield that farming season will be poor. But if a farmer has experienced workers around to give advice, then it will work well and it is a good way to help the soil fertility” (female-only focus group participant 2, Imo state)*

Another institutional barrier highlighted in the PCA result relates to credit unavailability. The interview findings confirm the credit constraint situation in the area and how it challenges the adoption of SLMPs. For example, P20 reported that because of the difficulty in getting a loan from the bank, he solely relies on getting interest-free loans from his Cooperative group, but the challenge is that:

“*the cooperative doesn’t have enough money to cater to the demand of all its members, therefore they [cooperative heads] ration the loan. So, the amount of loan that I get is not enough to purchase inputs required to maintain my land*”.

Similarly, P22 stated:

“*I have been taught how to implement terracing to control erosion on my sloping farmland, but I have not been able to do so because it requires hired labour, and I don’t have the money. To make matters worse, I can’t get a loan to pay for labour”*.

#### Factor 4: Constraints Related to Land Property Rights

According to the PCA result, constraints related to land property rights such as the unavailability and inaccessibility of land, and land tenure insecurity deter farmers’ adoption of SLMPs. The qualitative findings corroborate the PCA result. For example, the interview result suggests that scarcity of land in the areas significantly reduces fallow periods thus constraining the adoption of land fallow practices. P21 stated: *“Long ago, I left my farmlands to fallow for 4 years or 3 years but as I don’t have much land now, I reduced the fallow period to two years. As a result, the land will not be that fertile and it negatively affects yields”*.

Additionally, the interview results suggested that tenure insecurity discourages investment in long-term SLMPs, such as terracing and agroforestry, as farmers lose their right to the land at the end of their tenancy, which is usually for a relatively short duration (between one to two years). According to P22: *“I planted some trees on my land, but I don’t plant trees on the rented land; because just after harvesting your crops you give the land back to the owner”*. Another land-related constraint highlighted in the PCA result is uncontrolled livestock grazing which threatens the practice of crop residue management. This finding is supported by the discussion from the focus group, where a farmer stated: *“The invasion of cattle into our farmlands is a major problem for farmers here. Cattle often wander in and destroy the crop* residues*”*.

### Mitigation Strategies to Overcome the Constraints to the Adoption of SLMPs

**Fig. 2:**
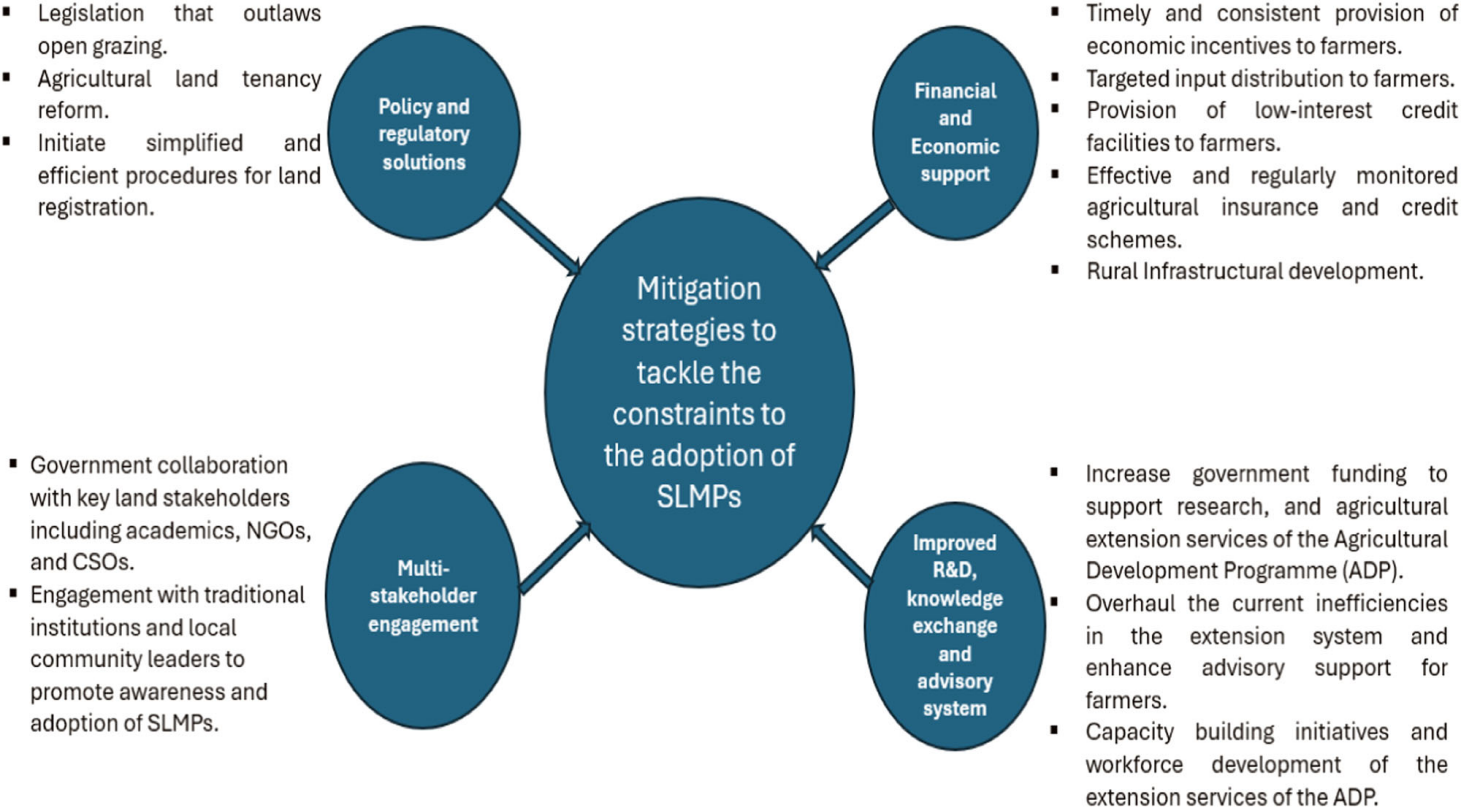
Overview of the mitigation strategies to tackle the constraints to the adoption of SLMPs based on the stakeholder’s perspective

Here we present the findings from the focus groups and interviews regarding the mitigation strategies to overcome the constraints to the adoption of SLMPs- research objective 2. The analysis of the qualitative data revealed five main mitigation strategies to tackle the constraints to the adoption of SLMPs. They include financial and economic support, improved R&D, knowledge exchange and advisory systems, policy and regulatory solutions, and multi-stakeholder engagement (Fig. 2).

#### Theme 1: Financial and Economic Support

Government provision of economic incentives to farmers to support them in various aspects of their farming activities emerged as one of the solutions to address the financial and economic challenges associated with adopting SLMPs. Participants emphasized that the government should timely provide free or subsidized agricultural inputs to farmers, targeted at their specific needs. From this perspective, P10 suggested: *“government should subsidise the cost of improved seedlings that shortens the harvest period of trees and ensure their distribution to farmers. This will encourage tree planting among farmers”*. The participants also emphasized that government support for any SLMP initiative communicates to farmers that the technology is profitable and offers tangible benefits. Therefore, they would show more interest in participating in the project. For example, P11 stated:

“*If you introduce any SLMPs to farmers along with inputs to support their implementation, the farmers will be motivated and encouraged to adopt. Also, the farmers reason that if the government can spend money on this project, there must be something about the project that will help us in our production, so they will readily adopt*”.

Advocating for targeted distribution of subsidised agricultural inputs to farmers, P3 stated: “*Before resource distribution by the government, there must be a field survey of the situation to reveal the immediate needs of the farmer - what kind of resources are needed and in what quantity*”. The participants further argued that effectively designed and regularly monitored agricultural insurance and credit schemes can be a key solution to the financial constraints faced by farmers, especially during the period they have to wait for the gains from investment in SLMPs to accrue. The participants emphasised the need for credit institutions to recognise the long-term nature of returns from certain SLMPs and factor this into the design of credit repayment plans. The participants also stressed the need to provide low-interest credit facilities to farmers, improve the loan application and approval process, as well as tackle the bureaucratic challenges in existing agricultural financing schemes such as Nigeria’s Incentive-Based Risk Sharing System for Agricultural Lending (NIRSAL) and the Anchor Borrower Programme. From this perspective, P12 stated that:

“*farmers often do not benefit from the available credit facilities which government boast of due to a lot of bureaucracy in the process. Often times the farming season ends before farmers receive the loan. When loans are received too late, there is a risk of misappropriation. Addressing these delays and simplifying the process is necessary to encourage farmers to apply for and effectively utilise loans*”.

Similarly, a focus group participants stated:

*“we [farmers] were asked to form cooperatives and apply for loan. We did so and applied and provided all the requirements, yet we did not get the loan. So, we are getting tired. So, it would be very helpful if something can be done to help poor farmers access the loan that government boast of” (mixed-gender focus group, participant 12, Anambra state)*.

P18 stated:

*“I prefer to get a loan from my cooperative because such loans are interest-free. If government wants to help farmers, let them provide low-interest loans and simplify the process of obtaining the loan”*.

Furthermore, government investment in rural infrastructure development, such as a good road network, was hailed as a comprehensive solution to improve the well-being of smallholder farmers in rural communities. The participants argued that such investment would allow easy movement of farmers to output and input markets, facilitate EAs’ visits to farmers’ plots, expand income opportunities for farmers, and ultimately alleviate farmers’ poor financial situation, which currently limits their adoption of SLMPs. For example, a male farmer stated during the focus group discussions:

“*Building good road networks that connect our farms to the main markets and nearby towns would enable us [farmers] to gain access to input and output markets. When we are connected to the market, we make sales and earn profits which we can use to invest in land management” (mixed-gender focus group participant 2, Imo state)*.

In the same vein, P15 stated: *that good road networks can result in more non-agricultural job opportunities, thus expanding income streams for farmers”*. P13 added: “*The condition of the roads leading to some farms is poor*, *which makes* *it difficult for EAs to visit farmers*”.

#### Theme 2: Improved R&D, Knowledge Exchange and Advisory System

The participants emphasized the necessity for increased national commitment to research and development in Nigeria, as well as the allocation of adequate funds to support the activities of research institutions, enabling them to conduct cutting-edge research to provide robust scientific evidence on the effectiveness of SLMPs. Moreover, the participants highlighted the need for capacity-building programmes, such as seminars, workshops, and training opportunities, focused on SLMPs. They proposed that these programmes should be initiated to develop the technical skills and expertise of researchers, academics, and other individuals directly or indirectly involved in addressing land degradation issues and promoting SLMPs. From this perspective, P1 suggested: *“I recommend* *investing in capacity building, especially for younger researchers, to provide them with the necessary training. Because without capacity building, we won’t be able to do cutting-edge research and projects that* *can lead to better policy formation”*.

Moreover, to mitigate the poor knowledge and lack of expertise in implementing and maintaining SLMPs among farmers, the participants emphasized the need for government funding of the extension service department of the ADP to overhaul the current inefficiencies in the extension system and enhance advisory support for farmers. From this perspective, P13 stated that: *“one important action to ensure sustained adoption of introduced SLMPs is ensuring EAs’ visit to farmers and particularly follow-up visits to check if they are farming as advised”*. Similarly, P14 advised: *“The state government should support the ADP by going back to the subvention system of funding, it will help us a lot to fund departmental activities and enable us to support the farmers in their activities concerning sustainable land management”*.

Moreover, the participants emphasised that capacity-building initiatives such as training programmes and workshops can help develop a skilled workforce capable of providing advice to farmers about SLMPs. On the other hand, P10 emphasized the urgent need for workforce development of the extension services to address the imbalanced extension agent-to-farmer ratio which has been a long-standing challenge to extension service delivery in Nigeria. According to P10: “*the high ratio of farmers to extension agents has been one of our major problems. We need more hands to effectively serve the farmers. The government should employ more extension agents and ensure regular payment for the existing staff”*.

#### Theme 3: Policy and Regulatory Solutions

Participants recommended that legislation that outlaws open grazing can help address the uncontrolled grazing of livestock which deters adoption of crop residue management in the area. Furthermore, the participants emphasised that open grazing that had led to the farmer-herder clashes not only affected their adoption of SLMPs but also threatened their security. Thus, abolishing open grazing would enable farmers to move freely around their farms without fear of being attacked by herders. Some participants further suggested that the ban on open grazing would help mitigate the challenges related to poor access to extension agents in the area. From this perspective, P9 cited: “Due to *the frequent herder-farmer clashes, farmers are afraid that somebody will come and attack them on their farms. When extension agents visit to demonstrate SLMPs, farmers feel afraid and often perceive them as a threat*”.

Furthermore, participants urged the government and policymakers to collaborate with community leaders to address land tenure insecurity issues within the study area. They suggested encouraging landlords to extend the duration of farmland leases and establish more formal and defined tenancy agreements to protect the rights of tenant farmers. From this perspective, P17 stated: *I think there is a need to improve the conditions for renting land. Sometimes, I am not sure whether the landlord would renew my tenancy after it expires. A more concrete arrangement between the farmer and landowner, recognised by the community leaders can help to solve this uncertainty in land tenure*”. On the other hand, the participants suggested that initiating simplified and efficient procedures for obtaining necessary land documentation can help to address tenure insecurity challenges for landlord farmers operating on land without formal documentation.

#### Theme 4: Multi-stakeholder Engagement

To increase awareness and enable the widespread adoption of SLMPs among farmers, the participants suggested that the government work in synergy with key land stakeholders including non-government organisations, and civil society groups. For example, P5 stated that: *“the intervention of NGOs, CSOs and then, of course, the private sectors which* have the necessary resources, *is important to promote SLMPs among farmers. These stakeholders should* play a key role in driving the initiative to *help farmers to adopt SLMPs. I don’t think the government can successfully handle it all alone”*. The participants also suggested that the government can leverage the close and trust-based relationship between local community leaders and the farmers to effectively promote awareness and adoption of SLMPs in the study area. P4 stated: *“There is a need for us to keep advocating and creating awareness of the best land management practices, especially to the traditional rulers, the palace secretary, and their cabinet. Because these traditional authorities are important opinion leaders - when they talk, their people listen to them”*.

## Discussion

This study examined the barriers to the adoption of SLMPs, and secondly investigated stakeholders’ perspectives on the strategies to tackle the adoption barriers. Regarding the first research objective, the PCA result showed economic/financial constraints as the principal constraint to the adoption of SLMPs. This finding highlights the inadequate financial resources of the farmers in the study area as a significant obstacle to adopting SLMPs, indicating the need for poverty alleviation programmes or economic empowerment initiatives. Other barriers to the adoption of SLMPs in the area, according to the PCA result include constraints related to the characteristics of the SLMPs, institutional constraints and constraints related to land property rights.

Regarding the economic/financial constraints, this study’s results suggest that the sampled farmers in the area are constrained by the absence/inconsistent provision of incentives by the government, lack of finance and the high cost involved in implementing SLMPs. This result is plausible given that implementing SLMPs demands additional operational and maintenance costs (such as the costs of maintaining terrace structures and the extra labour required for weeding after implementation). Without economic incentives from the government, many poor smallholder farmers may not be able to afford the cost of implementing SLMPs without compromising household food security and welfare. Consequently, the financial requirements for implementing SLMPs could be a barrier for resource-limited farmers. Similar to this study’s finding, Rahman et al. (2017) study in West Java, Indonesia and eastern Bangladesh found that financial constraints limit farmers’ willingness to adopt agroforestry. In a case study of southern African farmers, Andersson and D’Souza (2014) linked the partial adoption of conservation agricultural technologies to the insufficient quantities of input packages available to farming households.

Additionally, consistent with the findings of Emerton et al. (2016), this study result suggests that the nature of certain SLMPs in terms of their high upfront cost and longer timelines to see returns on investment, has made such SLMPs unattractive to farmers. This finding is plausible, especially considering that the majority of the sampled farmers are smallholder farmers who earn meagre income from farming (Table 2) and may have a pressing need to satisfy immediate household demands. Consequently, the sampled farmers would prefer a quick return on their investment in land and might find it challenging to invest in SLMPs that do not yield immediate profits. In addition, the study found that scarcity of crop residues due to their competing use for other purposes challenges the adoption of crop residue management. This finding is similar to the findings of Autio et al. (2021) in southeast Kenya where farmers’ adoption of crop residues was restricted by plant material scarcity.

Additionally, this study found that the problem of pest infestation following the implementation of crop residue discourages the practice of crop residue management among farmers. A similar finding is reported by Pedzisa et al. (2015) in Zimbabwe, where farmers’ use of crop residues is hindered by pest infestation. This study’s finding increases the incentive to test the suitability of specific SLMP to local agroecological conditions. Notably, the performance of SLMPs is determined by their compatibility with the agroecological conditions of the area (Thornton et al. 2018). Therefore, interventions to promote the adoption of SLMPs must consider the scale dependencies that may affect the benefits of SLMP adoption.

Among the institutional constraints, this study’s findings highlighted farmers’ lack of access to information and limited access to advisory support to correctly implement SLMPs as barriers to adoption. These findings are also aligned with the prior studies conducted in Southern Africa, Malawi, and Ethiopia (Cholo et al. 2018; Toth et al. 2017; Thierfelder et al. 2013). The reasons can be associated with the fact that the benefits of certain SLMPs are often dependent on the execution of crucial management actions (such as timely and appropriate fertiliser application, and training on installing terraces), farmers who lack this knowledge and expertise may find it difficult to successfully implement SLMPs. For example, Thierfelder et al. (2013) study in Southern Africa found that the lack of technical information on the management of manure and fertilizers and imperfect knowledge of the proper methods for spraying herbicides led to weed infestation, which ultimately discouraged adoption. Moreover, a study on the constraints to the adoption of fodder-tree technology in Malawi cited a general lack of information regarding the technology as the main constraint to adoption (Toth et al. 2017).

This study also revealed that the lack of access to credit presents challenges for the adoption of SLMPs in the study area, which are also highlighted in prior studies conducted in Nigeria (e.g., Olumba et al. 2023; Tankari 2015). The credit challenges can be associated with the cumbersome application process for obtaining credit from banks. The qualitative interviews revealed that farmers are required to complete various forms and provide collateral to apply for credit. Moreover, the credit takes a long time to be approved, which often means that loans arrive late, sometimes when the farming season is over. These inherent challenges in accessing credit from formal institutions reduce farmers’ enthusiasm for applying for formal credit; hence they are forced to rely on loans from individuals who either charge very high interest rates or cannot provide a sufficient amount of loan capital.

Additionally, this study result highlighted constraints related to land property rights including land tenure insecurity, limited availability and accessibility to agricultural land, and uncontrolled livestock grazing. Land is an important agricultural production resource and secure land tenure is critical for the adoption of SLMPs, particularly practices with longer payback periods such as agroforestry (Goldstein and Udry 2008; Olumba et al. 2024b; Rahman et al. 2017). The land tenure security issues in the area can be linked to the poor rate of land documentation, short lease duration and the existence of informal tenancy contracts in the study areas, which makes farmers uncertain about their right to the land in the next farming season but also exposes them to eviction from the land at the discretion of the landowner (Olumba et al. 2024a). Ranjan et al. (2019) demonstrate that annual lease renewals make farmers feel insecure and uncertain about their land tenure, making them doubtful about the likelihood of benefiting from their investment in SLMPs.

Regarding the land access and availability challenges in the area, insights from the focus group suggest that this challenge is caused by several factors including the high cost of available land and the loss of fertile agricultural land to erosion problems in the area. Okereke and Emeribeole (2020) also reported the reduction in available land for cultivation in southeast Nigeria resulting from soil erosion problems. Another study conducted in southwest Nigeria found that the conversion of farmland to residential or other construction purposes contributes to land availability challenges for farmers (Onanuga et al. 2022). Additionally, the study of Ihemezie et al. (2019) confirms a trend of agricultural land-use change in Benue state, Nigeria which is leading to a reduction in agricultural lands. Moreover, this present study found that uncontrolled livestock grazing threatens the practice of crop residue management. This finding highlights the growing concern over herder-farmer conflicts in Nigeria due to constrained access to land and water resources during grazing (Dimelu et al. 2017). Moreover, this finding underscores the need for action to enforce local rules and norms regarding livestock grazing without the farmers’ consent.

Regarding the second research objective, this study’s findings revealed several recommendations to tackle the constraints to the adoption of SLMPs, which were categorised as financial and economic support, improved R&D, knowledge exchange and advisory system, policy and regulatory solutions, and multi-stakeholder engagement.

The participants urged the government to consistently and promptly provide farm input subsidies to support farmers’ adoption of SLMPs. Furthermore, it should design effective agricultural insurance and credit schemes, in addition to establishing regulatory bodies to monitor the schemes’ implementation. Supporting the view of this study participants, Bassey and Uwadinma’s (2022) study highlights the significance of agricultural crop insurance for farmers and further suggests effective ways to encourage farmers’ participation in crop insurance schemes and develop a well-functioning insurance market. Reuben et al. (2020) study concludes that the agricultural credit guarantee scheme positively contributes to agricultural productivity in Nigeria.

Furthermore, this study’s findings highlight that the government needs to provide adequate infrastructure for rural areas such as good road networks. Rural roads in many developing countries, including Nigeria are in deplorable conditions, despite the significance of rural roads to the rural economy (Okeke and Nwankwo 2017). In line with the perspectives of this study participants, Olagunju and Akinbile (2020) argue that the provision of functional rural infrastructure and social amenities like a good road network can foster socio-economic development in rural areas and also improve the welfare of the farmers.

Additionally, the study participants urged the government to provide ring-fenced funding to support research institutions to conduct cutting-edge research, which will inform the design of evidence-based policies for scaling up SLMPs. The same view is expressed by Long et al. (2016) who recommended building the capacity of institutions and their personnel as a key step in scaling up new technologies and practices in agriculture. The government also needs to allocate ring-fenced funds to support the activities of the agricultural extension services of Imo and Anambra states ADP, including re-training of EAs to obtain new professional skills and competencies. Antwi-Agyei and Stringer (2021) concur that such actions can help improve the effectiveness of agricultural extension services, and potentially resolve the lack of knowledge and expertise in SLMPs among farmers.

The policy and regulatory solutions proposed by this study participants include actions to tackle land tenure insecurity issues such as facilitating the land documentation process, formalising land property rights and tenancy contracts and enacting legislation that outlaws open grazing. In line with the perspective of the participants, Edeh et al. (2022) argued that well-designed policies aimed at facilitating land documentation are critical in reducing land-related disputes and upholding individual land rights. Moreover, this study participants’ views on enacting legislation to outlaw open grazing corroborate other studies that argued that banning open grazing and establishing ranches following international best practices can help to curb the conflict between herders and farmers in Nigeria (Ugbudu 2021; Ugwueze et al. 2022). However, in contrast with the perspectives of this study participants, Balarabe (2021) argued that the adoption of legislation to prohibit open grazing may end up creating more problems than solutions. The scholar argues for the establishment of grazing routes to ensure both crop protection for farmers and respect for the rights of nomadic Fulani pastoralists. Furthermore, the study participants urge the government, policymakers, and community leaders to collaborate in designing tenancy contracts to address land tenure insecurities faced by tenant farmers. The collaboration between formal and local institutions to resolve land tenure insecurity is especially important given the pluralistic tenurial regime existing in Nigeria (Edeh et al. 2022). In formulating the tenancy reform, policymakers should examine successful agricultural tenancy contracts in advanced economies and develop appropriate frameworks and policies (Cowap 2021).

Finally, this study’s findings revealed multi-stakeholder engagement as a potential strategy to enhance the awareness of the benefits of SLMPs and subsequently farmers’ adoption of SLMPs. It involves effective collaboration between public and private sector organizations, non-governmental organizations, civil society groups, and traditional authorities. The study further emphasised the need to leverage the trust-based relationship between local community leaders and farmers. Local leaders often have deep-rooted connections, cultural understanding, and strong influence within their communities. Therefore, working with the local leaders ensures that SLMPs are not only adopted but also sustained over the long term. Corroborating the participant’s view, Akhtar‐Schuster et al. (2011) argued that multi-stakeholder collaboration is one of the promising approaches to promote SLMP initiatives to ensure successful outcomes. Similarly, Yiridomoh et al. (2022) noted the need for land sector agencies to work with traditional authorities to develop and implement context-specific and appropriate strategies to support SLMP adoption and implementation.

## Conclusion

Despite growing awareness of the potential of SLMPs to limit the degradation of agricultural and ecological systems and enhance farm yields, there is a notable lack of research focused on the constraints to the adoption of SLMPs, and the mitigation strategies to tackle the adoption barriers, especially in developing countries. Using a case study of 480 sampled farmers in erosion-prone areas of southeast Nigeria, this study aimed to investigate the challenges that farmers encounter when trying to implement SLMPs, and secondly examine stakeholders’ perspectives on the strategies to address the complex challenges to the adoption of SLMPs. This study’s results highlighted economic/financial factors as the principal constraint to the adoption of SLMPs. This suggests the need to mobilise significant financial resources from all sources (private and public) considering all levels (national to sub-national) to finance sustainable land management and provide adequate economic incentives to farmers to implement SLMPs. Other constraints to the adoption of SLMPs in the area include constraints related to the characteristics of the SLMPs, institutional constraints and constraints related to land property rights. The study findings highlight the necessity for collaborative work between formal and local institutions to resolve land tenure insecurity and improve access to credit by streamlining the associated bureaucracy.

Furthermore, the result of the qualitative analysis makes an important addition to the sustainable land management and land degradation literature by providing insights into the possible strategies for mitigating the challenges to the adoption of SLMPs. Specifically, the result revealed important mitigation strategies, categorised into four thematic areas: 1. financial and economic support, 2. improved R&D, knowledge exchange and advisory system, 3. policy and regulatory solutions, and 4. multi-stakeholder engagement. The findings of this study are important for the design of policies promoting the adoption of SLMPs and the restoration of degraded agricultural land, thereby enabling the achievement of target 15.3 of the United Nations SDG 15 on life on land. More generally, this study’s findings provide valuable analytical frameworks that can be applied to other developing economies experiencing similar constraints to adopting SLMPs.

## Supplementary information

Appendix constraints

## Data Availability

The datasets generated during and/or analysed during the current study are not publicly available due to confidentiality issues but are available from the corresponding author on reasonable request.
