# Supplementary material for: Investigating the Constraints and Mitigation Strategies for the Adoption of Sustainable Land Management Practices in Erosion-prone Areas of Southeast Nigeria
Source: Environ Manage. 2025 Jan 15;75(6):1504–19. doi: 10.1007/s00267-024-02104-y (PMC12084169; doi:10.1007/s00267-024-02104-y)
Supplement: Supplementary file 1 — Appendix constraints [file 267_2024_2104_MOESM1_ESM.docx]

**[Environmental Management](https://www.springer.com/journal/267)**

**Investigating the constraints and mitigation strategies for the adoption of Sustainable Land Management Practices in erosion-prone areas of Southeast Nigeria**

***Cynthia Nneka Olumba^1^, Guy Garrod^2^, and Francisco Areal^3^**

**School of Agriculture, Food & Rural Development, Newcastle University, Newcastle-upon-Tyne, United Kingdom.**

***Corresponding author’s email:** [**onunkacynthia@gmail.com**](mailto:onunkacynthia@gmail.com)**^1^;**

[**Guy.garrod@newcastle.ac.uk**](mailto:Guy.garrod@newcastle.ac.uk)**^2^;** [**Francisco.Areal-Borrego@newcastle.ac.uk**](mailto:Francisco.Areal-Borrego@newcastle.ac.uk) **^3^**

**Appendix 1: Multi-stage sampling procedure**

In the first stage, Anambra and Imo states were purposively selected from the five states in the Southeast region of Nigeria. The selected states were chosen based on their experiences of massive land degradation problems associated with inappropriate land management practices (Ndulue et al., 2021). In the second stage, two local government areas (LGAs) were randomly selected from each of the three senatorial zones in Anambra and Imo states, giving a total of twelve LGAs. The third stage involved the random selection of four autonomous communities from each of the selected twelve LGAs, giving a total of forty-eight autonomous communities. In the last stage, 10 farmers were selected in each of the selected communities to make a total of 480 respondents. Since the list of farmers in the communities was unavailable, we adopted a method employed in other studies to obtain a representative sample in the absence of a formal sampling frame (Kangogo et al., 2021). In this study, to select the 480 farmers, we interviewed one farmer per household, then skipped the next two households before surveying the third available household.

To achieve a random sample, at the second stage, for instance, the names of the LGAs in the senatorial zones were written down and numbered consecutively from 1 to n (n=17 for Imo state and n=21 for Anambra state). Then a list of random numbers was generated in Excel. The random numbers generated were then matched to the corresponding LGAs; any LGAs so identified were then selected.

**Appendix 2:** **Questionnaire**

**Section 1: Farm Characteristics.**

1. What is the total number of farmlands owned by the farmer? ___________________
2. What is the total size of all your farms? ________________
3. What is the measurement of your farmland? (1) Plots (2) Acres (3) Hectares
4. What is the distance from your home to your main farm in driving minutes? __________________
5. What is the distance from your main farm to the nearest market in driving minutes? ___________
6. How would you describe the condition of the road connecting your main farm to the market? (1) All-weather (2) Fair (3) Bad
7. Do you have any formal or informal tenure documentation attesting to use/ownership of your farmland? (1) Yes (2) No
8. How do you perceive the slope of your farmland? (1) flat (2) moderate/gentle slope (3) steep slope
9. How do you perceive the fertility of your farmland? (1) Poor fertility (2) Moderate fertility (3) Good/ very fertile.
10. Is any of your farmland suffering from soil erosion problems? (1) Yes (2) No
11. What is the tenure status of your main farmland? (1) Communal land (2) Sharecropped land (3) Rented land (4) Private/purchased land (5) Inheritance (6) Farming on land for free
12. Where is your farm located (1) Imo state (2) Anambra state?

**Section 2: Institutional factors and environmental awareness of farmers**

1. Are you a member of any village group or cooperative society? (1) Yes (2) No
2. How many times have you had contact(s) (face-to-face or phone) with extensions agents in the last year? ________________
3. Do you perceive any changes in climate patterns (rainfall or temperature) over the last 5 years? (1) Yes (2) No

**Section 3: Behavioural factors**

For Q17-Q20: Please indicate to what extent do you agree or disagree with the following statements?

1. I am someone who generally is fully prepared to take risks. (1) Strongly disagree (2) Somewhat disagree (3) Neither agree nor disagree (4) Somewhat agree (5) Strongly agree
2. I do not like to take risks in trying improved/newly introduced farming practices that are not used in the village. (1) Strongly disagree (2) Somewhat disagree (3) Neither agree nor disagree (4) Somewhat agree (5) Strongly agree
3. I am someone who generally is patient and willing to wait for future benefits. (1) Strongly disagree (2) Somewhat disagree (3) Neither agree nor disagree (4) Somewhat agree (5) Strongly agree
4. I believe that implementing SLM practices improves soil fertility and addresses soil erosion problems (1) Strongly disagree (2) Somewhat disagree (3) Neither agree nor disagree (4) Somewhat agree (5) Strongly agree

Display This Question:

If answer to ‘How many times have you had contact(s) (face-to-face or phone) with extensions agents in the last year??’ is not 0

1. I trust the information you receive from the extension agents. (1) Strongly disagree (2) Somewhat disagree (3) Neither agree nor disagree (4) Somewhat agree (5) Strongly agree

**Section 4: Adoption of SLMPs**

1. Do you plant trees or deliberately leave trees on your farmland? (1) Yes (2) No
2. What do you do to the plant materials (e.g leaves, stalks, roots) that remain after harvesting your crops? Please select all that applies. (1) Yes, I leave them on my farm to help soil fertility and/or control erosion (2) No I sell them (3) No I use them for firewood/ or fuel for cooking (4) No I burn them on the farm (5) No I use as livestock feed.
3. Do you leave your farmland uncultivated for one or more planting seasons to restore soil fertility? (1) Yes (2) No
4. Do you cultivate your land in such a way that reduce the amount you disturb the soil? (1) Yes (2) No
5. Do you use an integrated approach that combines the application of chemical fertilizers and organic manure to restore soil fertility? (1) Yes (2) No

Display This Question:

If answer to ‘How do you perceive the slope of your farmland?’ is not flat

1. In your farmland that is slopy, do you plant across the slope (horizontal) rather than up and down (vertical)? (1) Yes (2) No

**Section 5: Constraints to the Adoption of SLMPs**

1. To what extent do the following issues constrain your adoption of SLMPs?

| Constraints | very great extent | great extent | low extent | very low extent | NA |
| --- | --- | --- | --- | --- | --- |
| High prices of agricultural inputs (e.g., herbicides, fertilizers, manure) |  |  |  |  |  |
| Untimely/inconsistent supply of inputs by the government |  |  |  |  |  |
| Lack of economic incentives in terms of government support for agricultural input subsidies (seed and fertilizer), and farm implements. |  |  |  |  |  |
| High cost of SLMPs materials (e.g manure or improved seed varieties) |  |  |  |  |  |
| High cost of labour |  |  |  |  |  |
| Lack of finance to implement SLMPs |  |  |  |  |  |
| Failure of previous SLMPs implemented |  |  |  |  |  |
| Temporal delays in realising the benefits of SLMPs |  |  |  |  |  |
| Competing use of mulch materials/crop residue for livestock feed or firewood |  |  |  |  |  |
| Time demands of implementing SLMPs |  |  |  |  |  |
| Use of mulch materials/ crop residue could attract pests/rodents/mice to my farm |  |  |  |  |  |
| Lack of access to SLMPs information |  |  |  |  |  |
| Lack of access to credit from formal/informal financial institutions at the time needed and in sufficient quantities |  |  |  |  |  |
| Lack of access to technical assistance from extension agents |  |  |  |  |  |
| Lack of access to land at the time needed and in sufficient quantities |  |  |  |  |  |
| The fear that the land can be taken from you at any time |  |  |  |  |  |
| Uncontrolled grazing by livestock that feeds on crop residue on farm |  |  |  |  |  |

**Section 6: Socio-economic characteristics**

1. What is the gender of the respondent? (1) male (2) female
2. What is the gender of the household head? (1) male (2) female
3. What is the marital status of the farmer (respondent) ? (1) Married (2) Widowed (3) Single
4. What is the number of persons in the household feeding from the same food basket in the last 6 months? ______________________
5. How many years did the farmer spend in school? _____________
6. What is the age of the farmer? ______________
7. How long have the farmers been farming (in years)? _____________
8. What was your total farm income in the last farming season (naira)?
9. Is the farmer engaged in any non-farm work that helps to earn additional income? (1) Yes (2) No
10. Did you receive any money as a gift from anyone (family or friend) in the last one year? (1) Yes (2) No
11. How do you access credit/loan for your farming business? (1) Formal institutions e.g micro finance banks (2) Informal institutions e.g cooperatives, friends, relatives (3) I need credit, but I do not have access to any credit sources (4) I do not need credit

**Appendix 3: Focus Group Sessions Conducted in this Study.**

Focus group participants comprised farmers still practising, or who had previously practised, SLMPs. The farmers were selected based on their availability and willingness to attend and were recruited through the assistance of the extension agents who keep in constant contact with the farmers in each community. The time and venues for the focus groups were determined by the extension agents (EAs) and in agreement with the farmers. The mixed-gender sessions lasted between 45 and 60 minutes while the women-only sessions lasted between 25 and 35 minutes. The expenses arising from participation in the focus group were alleviated by providing a cash incentive to each respondent to cover travel and lunch. Such gestures are expected to enhance the quality of rapport with participants. The focus group began with traditional greetings and asking opening questions about the welfare of the participants and their loved ones. This opening strategy was done to gain rapport with participants and to make them feel at ease and relaxed. Thereafter, the objective of the study was explained to the participants. During the discussion, probing questions and prompts were used to encourage participants to elaborate on their responses.

| State | | | FGD-type | Community | Number of focus group session(s) | Number of participants per focus group session | Participant number |
| --- | --- | --- | --- | --- | --- | --- | --- |
| Anambra | Mixed gender | | | Nibo and Akpo communities | 2 | 5 males and 5 female farmers | 1-20 |
|  | | Women-only | | Nibo and Akpo communities | 2 | 5 female farmers | 1-10 |
| Imo | | Mixed gender | | Amatta community | 1 | 5 males and 5 female farmers | 1-10 |
|  | | Women-only | | Amatta community | 1 | 5 female farmers | 1-5 |
|  | |  | |  | 6 |  |  |

**Appendix 4:**  **Reflection on the Codebook Generation**

The data analysis and the codebook generation were based on an inductive thematic analysis of data to reflect the respondent’s views and experiences. As a first step, I read the transcript to familiarise myself with the data. During this process I made notes of any meaningful texts in the data in relation to my research questions. I started the process of the codebook development by generating preliminary codes. The coding process involved identifying important sections of transcripts that were meaningful within the context of my research question: what mitigation strategies can potentially tackle the constraints to the adoption of SLMPs? I then assigned as many different codes/labels as they fit to the several texts identified. The next stage was to organise the codes generated into themes based on the similarities and differences across the codes. Codes that share similar conceptual content were grouped in same category- themes, consequently four overarching themes were generated. I further reviewed the themes and codes within them, to ensure that the four themes were distinct from one another and the codes within them were relevant and reflective of the themes. The last stage of the analysis involved conceptualising the themes. The four themes were defined and assigned succinct phrases that adequately represent the codes that underpin them. Table A1 shows the codebook from thematic analysis of the data. Overall, the coding process was an iterative process supported by a continuous reflection on the research questions, and the transcripts data. This fostered reflexivity and improved the quality of the analysis such that the generated codes and themes accurately represents the data.

**Table A1: Theme generation codebook from thematic analysis of the data**

| **Themes** | **Description** |
| --- | --- |
| Financial and economic support | Provision of economic incentives to farmers to support their adoption of SLMPs. |
|  | Targeted input distribution to farmers. |
|  | Provision of low-interest credit facilities to farmers. |
|  | Effective and regularly monitored agricultural insurance and credit schemes to support farmers adoption of SLMPs. |
|  | Comments about infrastructural development in rural areas particularly road infrastructures to support farmers’ access to agricultural inputs and outputs markets and their engagement in off-farm employment to improve their economic status. |
| Improved R&D, knowledge exchange and advisory system | Recommendations to increase the funding to support research activities and agricultural extension services of the Agricultural Development Programmes (ADP). |
|  | The need to overhaul the current inefficiencies in the extension system and enhance advisory support for farmers. |
|  | Capacity building initiatives and workforce development of the agricultural extension services of the ADP. |
| Policy and regulatory solutions | Legislation that outlaws open grazing. |
|  | Agricultural land tenancy reform. |
|  | Initiate simplified and efficient procedures for land registration. |
| Multi-stakeholder engagement | Government collaboration with key land stakeholders including non-government organisations, and civil society groups to promote the adoption of SLMPs. |
|  | Engagement with traditional institutions and local community leaders to promote awareness and adoption of SLMPs. |

**References**

Kangogo, D., Dentoni, D., & Bijman, J. (2021). Adoption of climate‐smart agriculture among smallholder farmers: Does farmer entrepreneurship matter? *Land Use Policy*, *109*, 105666.

Ndulue DC, Ayadiuno RU, Mozie AT, Ndichie CC (2021) A Comparative Analysis of Soil Erosion Models for Tropical Humid of Southeastern Nigeria and Comparable Environments. Psychology and Education, 58(1): 5821-5835.
